# Calcium Influx and Male Fertility in the Context of the Sperm Proteome: An Update

**DOI:** 10.1155/2014/841615

**Published:** 2014-04-27

**Authors:** Md Saidur Rahman, Woo-Sung Kwon, Myung-Geol Pang

**Affiliations:** Department of Animal Science and Technology, Chung-Ang University, 4726 Seodong-daero, Anseong, Gyeonggi-Do 456-756, Republic of Korea

## Abstract

Freshly ejaculated spermatozoa are incapable or poorly capable of fertilizing an oocyte. The fertilization aptness of spermatozoa depends on the appropriate and time-dependent acquisition of hyperactivation, chemotaxis, capacitation, and the acrosome reaction, where calcium (Ca^2+^) is extensively involved in almost every step. A literature review showed that several ion channel proteins are likely responsible for regulation of the Ca^2+^ uptake in spermatozoa. Therefore, manipulation of the functions of channel proteins is closely related to Ca^2+^ influx, ultimately affecting male fertility. Recently, it has been shown that, together with different physiological stimuli, protein-protein interaction also modifies the Ca^2+^ influx mechanism in spermatozoa. Modern proteomic analyses have identified several sperm proteins, and, therefore, these findings might provide further insight into understanding the Ca^2+^ influx, protein functions, and regulation of fertility. The objective of this review was to synthesize the published findings on the Ca^2+^ influx mechanism in mammalian spermatozoa and its implications for the regulation of male fertility in the context of sperm proteins. Finally, Pathway Studio (9.0) was used to catalog the sperm proteins that regulate the Ca^2+^ influx signaling by using the information available from the PubMed database following a MedScan Reader (5.0) search.

## 1. Introduction

Spermatozoa are atypical cells with peculiar functionality: they are produced in one organism and released, and then they invade another organism and deliver their genetic material into a host cell to produce offspring by sexual reproduction. It is a well-known fact that only about 1 in 25,000 spermatozoa finally reaches the fallopian tube and gets the opportunity to fertilize an oocyte. In the mid-20th century, it had been claimed that mammalian spermatozoa are unable to fertilize an oocyte before achieving functional maturation, which occurs during their journey through the female reproductive tract for a finite period of time [1, 2]. This fundamental maturational process is chiefly regulated by numerous signaling cascades, and calcium (Ca^2+^) plays a dynamic role in this process, as an intracellular second messenger. Several studies have hypothesized that elevation of sperm intracellular Ca^2+^ ([Ca^2+^]_i_)/Ca^2+^ influx regulates motility, hyperactivation, chemotaxis, capacitation, and the acrosome reaction and facilitates the spermatozoa reaching and fertilizing of an oocyte [3–8]. Therefore, understanding the mechanism that regulates the Ca^2+^ influx in spermatozoa is a matter of utmost importance.

Previous studies have shown that the Ca^2+^ entry mechanisms are regulated via numerous Ca^2+^ permeable channel proteins in spermatozoa [6, 9, 10]. Therefore, the factors that regulate the functions of those channels will ultimately help us understand how male fertility is regulated. Recent applications of proteomic approaches such as two-dimensional polyacrylamide gel electrophoresis, mass spectrometry, and differential in-gel electrophoresis have yielded the identification of several sperm-specific proteins [11, 12]. These discoveries have provided new insight into protein functions and enabled us to recognize diverse sperm-specific processes in order to differentiate normal from abnormal spermatozoa [11]. Mature spermatozoa are widely known to be silent in both transcription and translation [11, 13, 14] or poorly capable of translation [15]; therefore, studies on individual sperm proteomes have described the importance of spermatozoal posttranslational modifications and their ability to induce physiological changes as a prerequisite for successful fertilization.

Torres-Flores et al. [16] have shown that human spermatozoa exposed to the phosphodiesterase inhibitor papaverine cause activation of protein kinase A (PKA) and stimulate the progesterone-induced Ca^2+^ influx via the cyclic adenosine monophosphate- (cAMP-) dependent pathway. Although these authors did not evaluate the relationship between* in vitro* fertility and Ca^2+^ influx, changes in intracellular pH and increased tyrosine phosphorylation ultimately provide a potential clue regarding sperm fertility competence. In another study, to evaluate hamster spermatozoa capacitation capability, comparative association was observed between pyruvate dehydrogenase A, Ca^2+^ influx, cAMP, and reactive oxygen species [17]. Additionally, Breitbart et al. [18] reported that polymerization of globular- (G-) actin to filamentous- (F-) actin occurs during capacitation. As capacitation and the acrosome reaction are Ca^2+^-mediated events [4, 5], one can, without considering further signaling cascade, assume that remodeling the actin structure might be linked with the regulation of Ca^2+^ influx in spermatozoa.

Recently, in our laboratory, we found that the manipulation of sperm proteins such as ubiquinol-cytochrome-c reductase core protein 2 (UQCRC2) [39], voltage-dependent anion channels proteins (VDACs) [4], and arginine vasopressin [5] could control the Ca^2+^ influx in spermatozoa and regulate capacitation, the acrosome reaction, and fertility. Therefore, design and construction of a similar study with most of the identified sperm proteins available from several protein databases might provide a more realistic insight into the Ca^2+^ influx, protein functions, and fertility. The present work reviews the latest information published by other laboratories as well as our research team on the aforementioned aspects of spermatozoa and their potential implications for diagnosis and prognosis of male fertility.

## 2. Mechanism of Ca^2+^ Influx in Mammalian Spermatozoa

The ultimate goal of fertilization of mammalian sperm is to fuse with and deliver their genetic materials into an oocyte [2, 40, 41]. For fertilization to occur completely, the spermatozoa must experience various obstacles both* in vitro* and* in vivo* [40, 41]. Ca^2+^ ions act as central signaling molecules; once they enter the spermatozoa, they exert allosteric regulatory effects on enzymes and many proteins [10, 21, 42]. Indeed, numerous elegant research findings have contributed significantly to our understanding of the molecular signaling of Ca^2+^ influx, especially through monitoring the activity of individual cells. However, most of the studies are discrete and often do not represent a cumulative idea. This section presents a compilation of some basic information regarding the Ca^2+^ entry mechanism into mammalian spermatozoa by recapitulating scientific evidence.

The literature reviewed shows that the primary source of Ca^2+^ for spermatozoa is the external environment: the fallopian tube in the female reproductive tract (*in vivo*) and culture media (*in vitro*) [8], and simultaneously increasing [Ca^2+^]_i_ regulates the release of Ca^2+^ into the cell. Therefore, how Ca^2+^ crosses into cells through the sperm plasma membrane is a matter of paramount importance. In eukaryotic cells, the Ca^2+^ influx occurs through specific Ca^2+^ permeable ion channel proteins located on the plasma membrane [43, 44] such as classical voltage-gated (high and low) Ca^2+^ (Ca_v_s), transient receptor potential (TRP), and cyclic nucleotide-gated (CNG) channels [9]. Recently, Ren and Xia have proposed four criteria to identify sperm ion channel proteins: detectability in sperm, preferably with knockout sperm as a negative control; ability to produce ion channel current detectable by patch-clamp recording; blocking of the channels that impairs normal sperm function; and mutations of gene encoding the ion channel proteins leading to sperm malfunctions [10].

**Figure 1:**
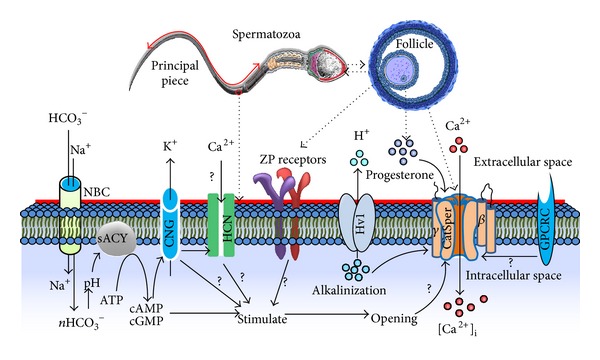
Possible signal transduction mechanisms of mammalian sperm Ca^2+^ influx through the Ca^2+^ permeable channel proteins. Previously published studies were used as references to summarize the list of channel proteins in spermatozoa. The channel proteins are localized mainly in the principle piece of spermatozoa. The follicular fluid and several factors in the fallopian tube (*in vitro* media) stimulate the receptors for spermatozoa Ca^2+^ influx. Ca^2+^ influx in spermatozoa is principally regulated by CatSper channels; however, the possible interaction between other channels that are responsible for controlling the opening of CatSper and allowing the Ca^2+^ into cells is indicated by arrow signs (red circle). The different channel proteins that are depicted in the diagram include the Na^+^-coupled HCO_3_^−^ transporter (NBC) family, soluble adenylyl cyclase (sACY), adenosine triphosphate (ATP), cyclic adenosine monophosphate (cAMP), cyclic guanosine monophosphate (cGMP), cyclic nucleotide-gated ion channel (CNG), hyperpolarization-activated cyclic nucleotide-gated channel (HCN), zona pellucida (ZP), the voltage-gated proton channel (Hv1), glutamate receptor family class-C (GPCRC), and an unknown mechanism (?).

The CatSper family of channels is the newest and only family of voltage-gated Ca^2+^ channels that meets most of the aforementioned criteria and essentially regulates Ca^2+^ entry into cells and is therefore crucial for sperm fertility [9, 45]. Four pore-forming CatSper channel proteins, CatSper 1–4, and at least two auxiliary subunits, CatSper*β* and CatSper*γ*, have been identified in a wide range of animals, including humans and mice [46, 47]. Physiologically, CatSper members are permeable to Ca^2+^,whereas the CatSper knockdown sperm does not have the channel current that is detected in the principal piece of wild-type sperm [20, 48]. Most of the channel proteins, including CatSper members, have been identified in the principal piece of spermatozoa [20, 46, 47, 49] (Figure 1). Although the explanation of such subcellular localization is still debated, it might be because of interactions among the channel proteins and with the auxiliary subunits, although a further study is needed to resolve this issue. Collectively, these proteins play a key role in various cellular processes via regulation of the membrane potential and intracellular ionic balance. Carlson et al. [50] and Quill et al. [51] have conclusively proved that CatSper1 and CatSper2 null mice are sterile owing to their inability to generate the sperm-hyperactivated motility prerequisite for penetration of an oocyte extracellular matrix. In effect, the complete or partial absence of single or multiple Ca^2+^ channels is responsible for infertility or subfertility, although their underlying signaling cascade has not been properly studied.

Previously, it has been reported that CatSper-dependent increases of [Ca^2+^]_i_ in spermatozoa are induced by several psychological stimuli such as cyclic nucleotides (e.g., cAMP and cGMP) [29, 30, 52], soluble adenylyl cyclase [29, 52], zona pellucida glycoprotein [34, 35, 38], serum albumin [37, 38], secretion of cumulus oophorus [38], intracellular alkalization [3, 53], and pH [6, 21]. A recent study showed that endocrine disruptors such as p,p′-dichlorodiphenyldichloroethylene (p,p′-DDE) promoted Ca^2+^ entry into spermatozoa by activating CatSper channels, even at a physiological concentration [36]. In addition, several other components are also known to play an important role in Ca^2+^ influx mechanisms in mammalian spermatozoa by regulating the opening of CatSper members, including the flagellar voltage-gated proton channel (Hv1) [21], Ca^2+^-ATPase pump [33], several cyclic nucleotide-gated ion channels (CNG) [27, 54], hyperpolarization-activated cyclic nucleotide-gated (HCN) channels [27], and G-protein coupled receptors (GPCRs).

A hypothetical signaling cascade of Ca^2+^ influx pathways and interaction of several channel proteins is depicted in Figure 1. Although the functions of several ion channel proteins together with their concurrent relationship with numerous stimuli have been well studied [21, 27, 38], several fundamental questions remain unanswered; for example, how do these channels/stimuli regulate the Ca^2+^ influx during spermatozoa processes such as capacitation, the acrosome reaction, and fertilization? Do they work alone or together with other channel proteins to regulate Ca^2+^ influx? Moreover, which other parameters that remain undetected could have an effect on Ca^2+^ influx? Therefore, future research should focus on resolving these issues.  Table 1 summarizes the proposed effect of Ca^2+^ ion channels and their physiological role that ultimately helps Ca^2+^ influx into mammalian spermatozoa.

## 3. Effect of Ca^2+^ Influx on Male Fertility

Ca^2+^ triggers multiple physiological events in spermatozoa, such as hyperactivation, chemotaxis, capacitation, and the acrosomal reaction, all of which are essential for successful fertilization. In mammalian spermatozoa, numerous Ca^2+^ permeable channel proteins control intracellular pH, and the pH-dependent Ca^2+^ influx is measured by the whole-cell patch clamp technique [9, 20]. A review of the literature showed that a potential functional interaction exists between the sperm proteins and Ca^2+^ permeable channel proteins, thus modulating the Ca^2+^ influx mechanism [4, 5, 39] and playing a vital role in adjusting male fertility. However, the mechanism by which Ca^2+^ triggers intracellular signaling to regulate physiological events in spermatozoa and the role of sperm proteins in adjustment of Ca^2+^ influx into cells remains unclear. This topic is emphasized below.

### 3.1. Ca^2+^ Influx, Sperm Hyperactivation, Chemotaxis, and Protein Functions

In general, mature spermatozoa are held immotile within the epididymis. However, they quickly begin to swim following release. This is known as activation of motility and is characterized by symmetrical flagellar beats [55, 56]. The terms sperm activation and hyperactivation have quite different meanings. The spermatozoa become hyperactivated when the amplitude of the flagellar bend increases and produces a highly asymmetrical beat.* In vivo*, hyperactivation of spermatozoa facilitates the release of sperm from oviductal storage and boosts them through mucus in the oviductal lumen and matrix of the cumulus oophorus during fertilization [7]. In contrast, chemotaxis is a form of sperm movement in which spermatozoa move toward a concentration gradient of a chemoattractant released from the oocyte [57, 58]. However, the molecular event that characterizes spermatozoa chemotaxis is only partially known [57].

**Figure 2:**
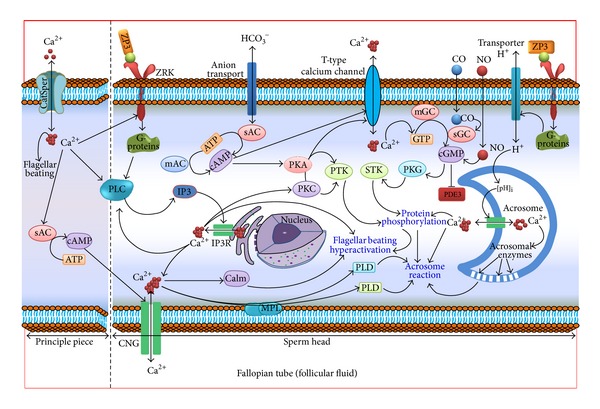
Schematic diagram showing the mechanism of Ca^2+^ regulated hyperactivation, capacitation, and the acrosome reaction of spermatozoa, which are three principal events of fertilization. Ca^2+^ together with ZP3 (zona pellucida glycoprotein-3) exhibits the most important role in sperm binding and acrosomal reaction. Ca^2+^ triggers the zona pellucida (ZP) receptors of cell membrane that activate G-proteins in the sperm head. Activated G-proteins stimulate the H^+^ transporter to increase intracellular pH, ultimately inducing the acrosomal reaction and hyperactivation by catalyzing the acrosomal enzymes [91]. Cyclic adenosine monophosphate (cAMP) and cyclic guanosine monophosphate (cGMP) are produced from adenosine triphosphate (ATP) owing to enzymatic catalysis by soluble adenylate cyclase (sAC) and guanylate cyclase (sGC), respectively, in mature spermatozoa. The bicarbonate ions activate the sAC; however, follicular fluid also stimulates the sAC through release of Ca^2+^ ions via the CatSper channel (principal piece). However, G-protein mediated signal transduction activates sAC and phospholipase-C (PLC) that ultimately causes tyrosine phosphorylation [51, 92], which is responsible for events such as capacitation and the acrosomal reaction. Likewise, extracellular signals such as nitric oxide (NO) and carbon monoxide (CO) stimulate membrane-bound GC (mGC) and sGC, respectively, to synthesize cGMP. Increases in cGMP level evoke a concomitant increase in cAMP by inhibiting its PDE3. However, the increased Ca^2+^ level can also directly catalyze cAMP [93, 94]. Activated sAC, sGC, and PLC stimulate the generation of the second messengers' inositol trisphosphate (IP3) like cAMP, cGMP. The IP3 binds to the IP3 receptor (IP3R) to increase [Ca^2+^]_i_ via the release of the [Ca^2+^]_i_ storage ions. Concurrently, the second messengers activate protein kinases (PKA, PKC, and PKG), in turn gating ions through the T-type calcium channels, cyclic-nucleotide gated ion channel (CNG), and so on, that together with the activation of protein tyrosine kinases (PTK) and serine/threonine protein kinase (STK) cause increased protein phosphorylation [93, 94]. Additionally, the CatSper Ca^2+^ activates calmodulin (Calm), phospholipase-A (PLA), and phospholipase-D (PLD) with increased generation of other second messengers during the acrosome reaction. Ca^2+^ influx together with increased protein phosphorylation brings about the capacitation response that is responsible for the waveform asymmetry of motility termed hyperactivation during fertilization. Both hyperactivation and the acrosomal reaction boost flagellar beating, ultimately resulting in the penetration of the outer egg coat and subsequent fertilization of the mature ovum [91–95].

There is strong evidence to support that sperm hyperactivation and chemotaxis are required for penetrating the zona pellucida [48, 57, 59, 60]. Incubation of spermatozoa with an extracellular Ca^2+^ source induces hyperactivation in mammalian spermatozoa [61, 62] and chemotaxis in starfish [57]. In addition, measuring cytoplasmic Ca^2+^ levels by using the fluorescent Ca^2+^ indicator indo-1 proved that spermatozoa hyperactivation is potentially regulated by Ca^2+^ influx. However, it is unknown whether Ca^2+^ influx independently induces hyperactivation/chemotaxis in mammalian spermatozoa. Ho and Suarez [56] proposed that sperm hyperactivation induced by Ca^2+^ influx is mainly pH-dependent because sperm require a pH of 7.9–8.5 for hyperactivation, whereas activation can occur at a pH < 7.0. The proposed model of Ca^2+^-induced hyperactivation is represented in Figure 2.

It has recently been found by our laboratory that treatment of mouse spermatozoa with nutlin-3a, a small molecule antagonist of the mouse double minute 2 repressor, potentially downregulates the functions of the ubiquinol-cytochrome-c reductase complex component UQCRC2 and correlated with significantly reduced [Ca^2+^]_i_ and sperm hyperactivation. This study provided insight that the Ca^2+^ influx in spermatozoa is partially regulated by UQCRC2 protein. Kwon et al. [4] reported that blocking VDAC with 4,4′-diisothiocyanostilbene-2,2′-disulfonic acid (DIDS) significantly decreased sperm hyperactivation. A significant decrease in [Ca^2+^]_i_ was observed in (−) DIDS conditions, while [pH]_i_ significantly increased in (−) DIDS, regardless of Ca^2+^. Simultaneously, a significantly elevated [pH]_i_ was observed in (+) Ca^2+^. This study provides strong evidence that the modulation of Ca^2+^ influx by VDACs is pH-dependent, which is consistent with the result of a previous study by Ho and Suarez [56]. Moreover, another study proposed that deamino [Cys 1, d-ArgS] vasopressin (dDAVP), an AVPR2 agonist, significantly decreased sperm motility and intracellular pH, but, interestingly, it increased [Ca^2+^]_i_ by regulating the function of arginine vasopressin in mice spermatozoa. However, it remains to be clarified as to why spermatozoa motility is decreased even in increased [Ca^2+^]_i_ conditions.

**Figure 3:**
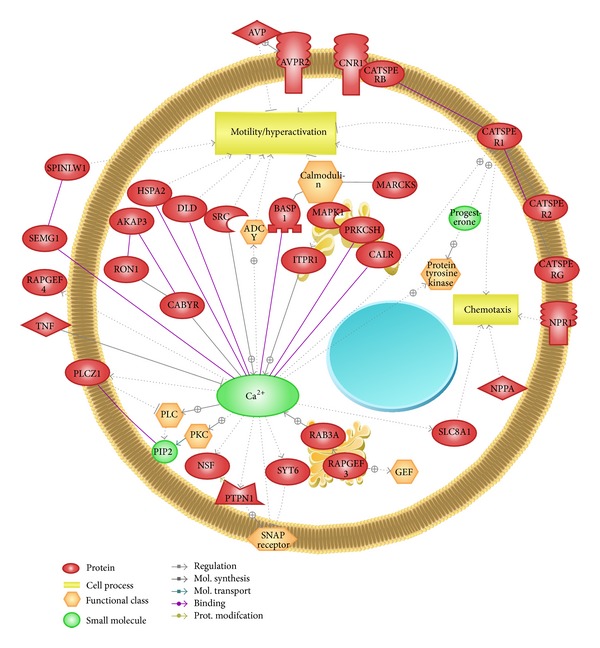
Schematic representation of interactions among ~35 proteins related to Ca^2+^ regulated spermatozoa hyperactivation and chemotaxis. The figure was produced by use of Pathway Studio (9.0) following the MedScan Reader (5.0) protein search from PubMed database [12].

On the basis of the findings of the aforementioned studies, it is tempting to hypothesize that spermatozoa hyperactivation is mostly controlled by Ca^2+^ influx. However, potential interactions exist between protein functions. Therefore, Ca^2+^ influx, protein interaction, and hyperactivation might give numerous different annotations of upcoming research in this field. We have illustrated a schematic representation of different signaling pathways involving sperm proteins by using Pathway Studio. These proteins exhibit significant modifications to induce sperm hyperactivation and chemotaxis in spermatozoa by regulating Ca^2+^ influx (Figure 3).

### 3.2. Ca^2+^ Influx versus Capacitation, the Acrosomal Reaction, Fertilization, and Sperm Proteome

Mammalian fertilization is a species-specific episode that is accomplished by a complex set of molecular events. To fertilize an oocyte, multiple extreme changes occur in spermatozoa that begin from its formation in the testes of the male reproductive tract to its penetration and fusion with an egg in the female reproductive tract. Although spermatozoa are motile as well as morphologically normal after ejaculation, they are unable to fertilize an oocyte [59]. They gain the fertilization ability only after educating in the female reproductive tract [40], and the modifications that spermatozoa experience during this time are collectively known as “capacitation.” Only capacitated spermatozoa can undergo the acrosome reaction through binding to the egg zona pellucida, and they finally become capable of penetrating and fertilizing the egg [4, 18, 39].

The term “capacitation” was proposed by Austin in 1952 [1], although this concept was initially described by both Chang and Austin in 1951 [2, 41]. In fact,* in vivo* capacitation takes place in the female reproductive tract; however, it is also possible to capacitate spermatozoa* in vitro* by using particular media containing appropriate electrolytes and pH [2]. In an elegant review, Visconti summarized that the early stage of capacitation mainly comprises the bicarbonate-mediated activation of sperm motility, whereas the late stages include intracellular alkalinization, increase in protein tyrosine phosphorylation, and preparation for the acrosomal reaction [63]. These temporal differences in capacitation and the acrosome reaction require numerous mechanisms, and Ca^2+^ influx plays a significant role in the process [63, 64]. Fraser [65] reported that capacitation is a comparatively slow event that requires several hours to complete and is mainly regulated by a modest rise in [Ca^2+^]_i_, whereas the acrosome reaction is an exocytosis process that occurs very rapidly (within a minute) and is triggered by a large influx of [Ca^2+^]_i_ [65, 66].

Although the biochemical phenomenon of Ca^2+^ regulated capacitation and the acrosome reaction have been known for the last two decades, the molecular basis of this process is still poorly understood. For capacitation, the cholesterol influx initially stimulates the elevation of [Ca^2+^]_i_ and bicarbonate into the spermatozoa and finally activates PKA and tyrosine phosphorylation, respectively, via the production of the cAMP [66–68]. In addition, binding to the zona pellucida causes additional activation of cAMP/PKA and protein kinase C (PKC) [68–70]. Spermatozoa need [Ca^2+^]_i_ influx to proceed further, and they are believed to be activated by PKC through the opening of the calcium channels. Interestingly, PKA together with a secondary messenger, inositol trisphosphate, activates calcium channels localized in the outer acrosomal membrane and increases the calcium concentration in the cytosol. Further increase of cytosolic Ca^2+^ influx occurs through a store-operated calcium entry mechanism in the plasma membrane, resulting in further depletion of Ca^2+^ in the acrosome [68, 69].

In support of the aforesaid studies, several recent studies on the same topic have also hypothesized that, after the morphological maturation of spermatozoa for sperm-oocyte fusion, [Ca^2+^]_i_ decreases because acrosome-reacted spermatozoa release a substantial amount of Ca^2+^ from their inner cell layers [71, 72]. Ca^2+^-mediated capacitation and the acrosome reaction have been illustrated in Figure 2 for better understanding. However, for a more in-depth understanding, we recommend reading some excellent reviews on this topic [63, 67, 73–77].

A review of the literature showed that several sperm proteins potentially regulate the Ca^2+^-dependent capacitation and the acrosome reaction in mammalian spermatozoa [4, 5, 39]. However, how these proteins regulate the Ca^2+^ influx in spermatozoa is a matter that remains to be elucidated. Breitbart et al. [18] reported that formation of F-actin mostly depends on PKA, protein tyrosine phosphorylation, and phospholipase D activation during capacitation. Ca^2+^ is one of the principle regulators of capacitation, and it is therefore tempting to hypothesize that organizational modification of F-actin in spermatozoa together with interacting with other sperm proteins has potential influence on Ca^2+^ influx. A similar finding has been established more precisely by another study [78], where boar sperm capacitation was studied by combined application of computational and experimental approaches. These authors reported that the boar spermatozoa capacitation network contains several connecting cascades, whereas only three nodes bound to all the subcellular compartments are involved in spermatozoa postejaculatory signaling, such as [Ca^2+^]_i_, ATP, and actin polymerization. Removal of the actin polymerization node from this aforesaid network causes disorganization of the network topography and affects capacitation, and this has been confirmed by zona pellucida-induced capacitation and the acrosomal reaction in an* in vitro* demonstration [78].

In another study, Patrat et al. [79] showed that progesterone (P_4_) that is secreted by cumulus cells directly acts on the sperm plasma membrane and triggers the intracellular signals and enzymatic pathways involved in the acrosome reaction. P_4_ regulates the acrosome reaction and is mediated by a compulsory Ca^2+^ increase. This study found that P_4_ induced the activation of Gi/Go protein-coupled and protein tyrosine kinase receptors, and it affected capacitation and the acrosome reaction. In contrast, Ca^2+^ regulated exocytosis of spermatozoa requires active acrosomal proteins such as N-ethylmaleimide-sensitive factor (NSF) [66]. Additionally, the same research team showed that the ras-related protein Rab-3A (RAB3A) is also necessary for Ca^2+^-dependent exocytosis. Interestingly, Rab3A activation of acrosomal exocytosis requires active NSF. Therefore, protein-protein interaction might also play a potential role in regulating Ca^2+^ influx. All of these observations seem to be consistent with the idea that Ca^2+^ functions are regulated by sperm proteins during fertilization. However, the key question is how do these proteins modify Ca^2+^ influx in spermatozoa?

**Figure 4:**
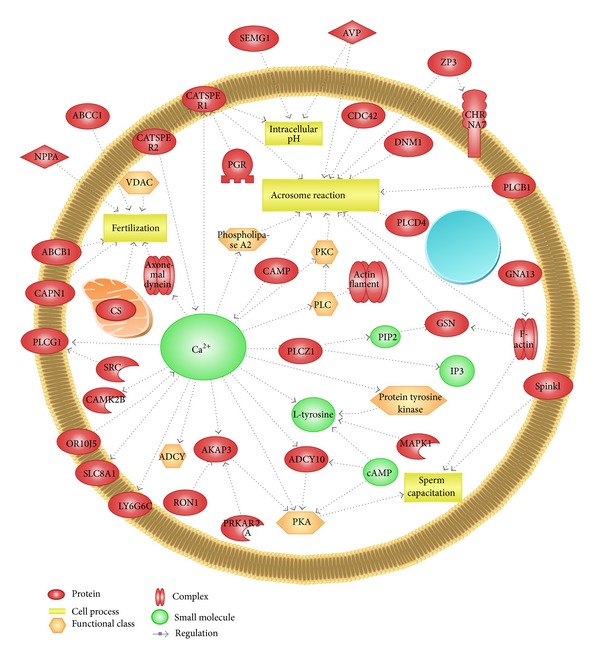
Schematic representation of interactions among ~40 proteins related to Ca^2+^ regulated spermatozoa capacitation, the acrosome reaction, and fertilization. The figure was produced by use of Pathway Studio (9.0) following the MedScan Reader (5.0) protein search from PubMed database [12].

Recently, in our laboratory, we used mice spermatozoa to evaluate the interrelationship of proteins related to Ca^2+^ influx, including UQCRC2 [39], arginine vasopressin [5], and VDACs [4], and evaluate their effects on capacitation and the acrosome reaction. It is likely that a sustained phase of Ca^2+^ is required for fertilization and might be regulated by the complex interaction of numerous sperm proteins. Therefore, studies to identify proteins that might have the ability to induce such a change are worth undertaking. Application of Pathway Studio helped us represent over 40 proteins that are potentially implicated in Ca^2+^ mediated regulation of capacitation, the acrosome reaction, and male fertility (Figure 4).

### 3.3. Ca^2+^ Influx and Postfertilization Egg Activation in Context of Sperm Proteome

Ca^2+^ influx in spermatozoa is not only important for sperm maturation, but it is also equally important for activation and development of the oocyte. A study of egg activation by Ca^2+^ was conducted by Steinhardt and colleagues in 1974 and showed remarkable findings [80]. Steinhardt et al. [80] reported that administration of Ca^2+^ ionophores induced the early events of hamster egg activation. Thus far, it has been shown that the eggs of almost all species are activated by an increase in Ca^2+^ oscillation by spermatozoa during fertilization [81, 82]. However, how the spermatozoa trigger the oocyte Ca^2+^ oscillation remains to be elucidated. Several hypotheses have been proposed to describe these mechanisms [83–86].

**Figure 5:**
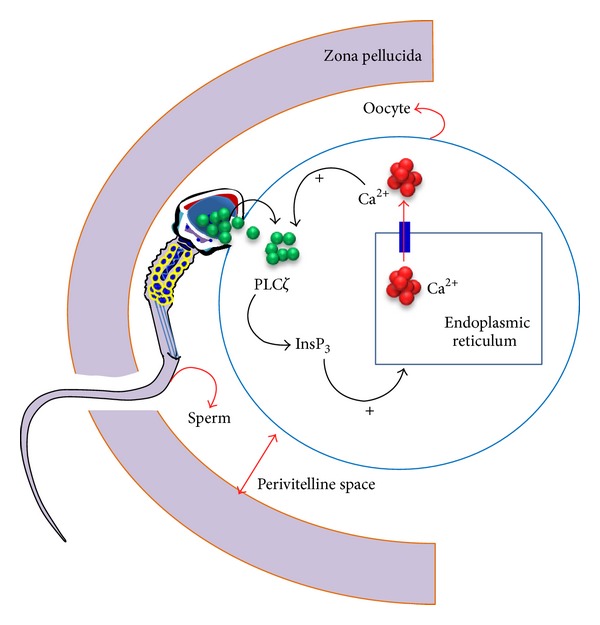
Schematic representation showing the Ca^2+^ influx mechanism in mammalian eggs stimulated by mature spermatozoa. Spermatozoa donate the phospholipase C isoform zeta (PLC*ζ*) protein within a few minutes of sperm-egg fusion (represented by green color circle). Inositol 1,4,5-trisphosphate (InsP_3_) is produced due to the hydrolysis of PLC*ζ*, which subsequently triggers the nsP_3_ receptor-mediated Ca^2+^ release (indicated by red color circle) from the endoplasmic reticulum of the oocyte. Simultaneously, the increased cytoplasmic Ca^2+^ leads to further PLC*ζ* stimulation, leading to the positive feedback loop of Ca^2+^ and InsP_3_ rise. The hypothesis has been modified from Swann [96] and Swann et al. [97].

**Figure 6:**
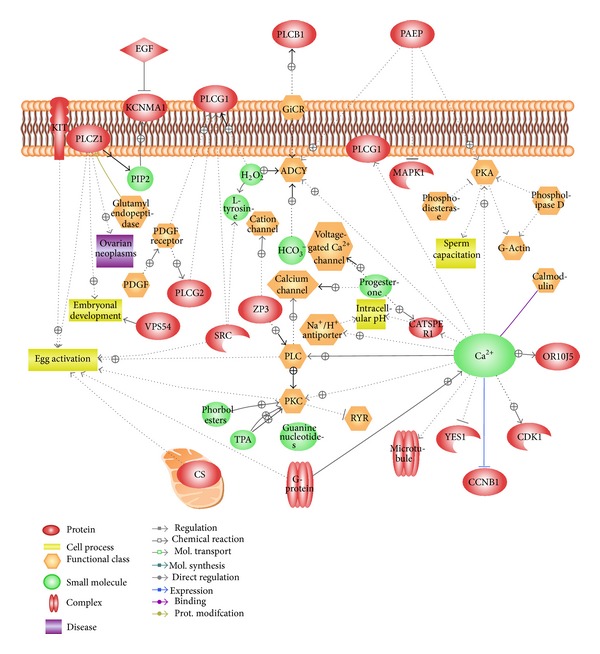
Schematic representation of interactions among ~30 proteins related to Ca^2+^ regulated egg activation and embryonic development. The figure was produced by the use of Pathway Studio (9.0) following the MedScan Reader (5.0) protein search from PubMed database [12].

It has been reported previously [83] that the spermatozoa introduce Ca^2+^ influx into oocytes by a specific protein called oscillogen in hamsters. Recent studies have shown that phospholipase C zeta (PLC*ζ*), a novel sperm-specific agent, is responsible for induction of Ca^2+^ oscillation in eggs after sperm-egg membrane fusion [87–89]. According to this mechanism, the sperm protein PLC*ζ* causes the release of [Ca^2+^]_i_ in eggs and is mediated via inositol 1,4,5-trisphosphate (InsP_3_) receptors (hypothetical depiction in Figure 5). Even when the InsP_3_ or its derivatives are injected into unfertilized, mature eggs, oscillation occurs due to the unique feedback properties of the InsP_3_ receptors in mouse eggs [90]. However, it is still unknown whether there are any other factors/proteins available in spermatozoa that also have similar effects. We illustrated the relevant signaling and metabolic pathways by using sperm proteins to facilitate the understanding of the mechanisms behind Ca^2+^ mediated activation of oocytes (Figure 6).

## 4. Future Prospects

The maturational events of mammalian spermatozoa are strictly regulated through the well-coordinated Ca^2+^ influx. It is the central regulator of many key activities in spermatozoa, all of which are necessary for fertilization. However, our current understanding at the molecular level concerning Ca^2+^ signaling in the spermatozoa is insufficient. Therefore, a better understanding of such an event can provide a more complete comprehension of Ca^2+^ regulated sperm functions and fertility optimization.

A large number of Ca^2+^ permeable ion channel proteins have been identified [10, 43] that collectively regulate the Ca^2+^ influx mechanism in spermatozoa. Although the recent application of patch-clamp recordings of channel current significantly improves our understanding of the functions of these channel proteins, several basic aspects remain unsolved, such as identifying the functions of individual channels in spermatozoa and how these channels coordinate Ca^2+^ influx. Therefore, production of knockdown animals and using them as negative controls compared with their wild counterparts might provide more specific ideas about channel functions. CatSper is the one of the well-studied channel proteins [10] and the functions of different pore-forming CatSper channels (1–4) and auxiliary subunits (CatSper*β* and CatSper*γ*) remain a matter to be elucidated.

A literature review found that the Ca^2+^ influx mechanism in spermatozoa is regulated by several physical stimuli, although the underlying mechanism is less clearly defined. Protein-protein interactions also potentially regulate the Ca^2+^ uptake mechanism in spermatozoa. Although recently applied proteomic approaches have identified several sperm-specific proteins, their functions in Ca^2+^ regulation and interaction with channel proteins are unclear. Therefore, future research should target this topic to provide a robust understanding of Ca^2+^ and male fertility in both humans and other animal species.

## Figures and Tables

**Table 1:** Summary of published works on ion channels and physiological stimuli of mammalian spermatozoa that regulate the Ca^2+^ influx mechanism.

Name of channel/stimuli	Localization on spermatozoa/availability	Role in Ca^2+^ influx	Role in sperm physiology	Effect of knocking down/absence	Reference
CatSper CatSper 1 CatSper 2 CatSper 3 CatSper 4 CatSper*β* CatSper*γ*	Principal piece	Regulates Ca^2+^ influx	Ca^2+^ uptake, hyperactivated motility	Sterile	Barratt and Publicover, [19]; Qi et al. [20]

Hv1	Principal piece	Intracellular pH, alkalization thus stimulate Ca^2+^ influx	Extrudes protons from flagella, alkalization	Fertile	Lishko et al. [21], Lishko et al. [22]

*I*_ATP_	Midpiece	Selectively transports the Ca^2+ ^	Ca^2+^ influx, alkalization	Fertile	Navarro et al. [23]

TRPC	Principal piece, midpiece	Stimulates opening of CatSper	Ca^2+^ influx, cell depolarization	Fertile	Gees et al. [24], Castellano et al. [25]

CNG	Sperm flagellum, head	Stimulates opening of CatSper via cAMP/cGMP	Ca^2+^ influx	Fertile	Biel and Michalakis [26]

HCN	Flagellum	Depolarization and opening of CatSper	Ca^2+^ influx	Fertile	Wiesner et al. [27]

SOC	Plasma membrane	ZP-induced Ca^2+^ influx	Sperm chemotactic	Subfertile	Yoshida et al. [28]

sACY cAMP/cGMP	Intracellular space and cell membrane	Activates CatSper, CNG, and HCN to regulate Ca^2+^ influx	Ca^2+^ influx, alkalization	Sterile	Esposito et al. [29], Hess et al. [30]

GPCR(s)	Principal piece, midpiece	ZP-induced Ca^2+^ influx increases in [Ca^2+^]_i_	Maintains fertilization	Subfertile	Fukami et al. [31] Fukami et al. [32]

PLC*δ*	Acrosome	ZP induced increases in [Ca^2+^]_i_	Ca^2+^ influx	Subfertile	Fukami et al. [32]

Ca^2+^-ATPase pump	Principal piece	Intracellular pH and alkalization	Ca^2+^ influx, capacitation	Motility loss results in infertility	Wennemuth et al. [33]

ZP glycoproteins	Follicle	Induced Ca^2+^-dependent increase in [Ca^2+^]_i_	Hyperactivation, capacitation	Delayed capacitation	Florman [34], Florman et al. [35]

Endocrine disruptor (p,p′-DDE)	Female reproductive tract	Activates CatSper	Ca^2+^ influx	Motility loss, delayed capacitation	Tavares et al. [36]

BSA	Extracellular space	Similar to ZP glycoprotein	*In vitro* capacitation	Motility loss, subfertility	Xia and Ren [37] Bailey and Storey [38]

Oviductal and follicular fluid	Extracellular space (*in vivo*)	Ca^2+^-dependent increase in [Ca^2+^]_i_ in sperm	Ca^2+^ influx	Motility loss delayed capacitation	Xia and Ren [37]

Hv1: voltage-gated proton channel; *I*_ATP_ *: *ATP-gated channel; TRPC: transient receptor potential channels; CNG: cyclic nucleotide-gated ion channel; HCN: hyperpolarization-activated cyclic nucleotide-gated channel; SOC: store-operated Ca^2+^ channel; cAMP: cyclic adenosine monophosphate; cGMP: cyclic guanosine monophosphate; sACY: soluble adenylyl cyclase; GPCR: glutamate receptor family class-C; PLC*δ*: phospholipase C zeta; ZP: zona pellucida; p,p′-DDE: p,p^'^-dichlorodiphenyldichloroethylene; BSA: bovine serum albumin.
